# Global research trends in DNA methylation in rheumatoid arthritis: A bibliometric analysis and visual analysis

**DOI:** 10.1097/MD.0000000000036218

**Published:** 2024-01-05

**Authors:** Xin Huang, Longxiang Huang, Xiang Gao, Changhua Liu

**Affiliations:** a Chengdu University of Traditional Chinese Medicine, Chengdu, Sichuan Province, China.

**Keywords:** autoimmune diseases, bibliometric analysis, DNA methylation, rheumatoid arthritis

## Abstract

Rheumatoid arthritis (RA) is a prevalent autoimmune disorder with a significant global economic burden. Epigenetic modifications, particularly DNA methylation, play a crucial role in RA. This study conducted a bibliometric analysis to explore the evolving trends and predominant themes in RA and DNA methylation research over the past two decades. A total of 1800 articles met the inclusion criteria, and the analysis revealed consistent growth in the literature, with a notable increase in output after 2019. The research involved 70 countries, 2139 academic institutions, 23,365 unique authors, and 58,636 co-cited authors. The United States emerged as a dominant contributor in this research domain. The significance of DNA methylation in shaping research directions for RA management is increasingly evident. Recent investigations have shed light on the pivotal role of DNA methylation in RA, particularly in characterizing synovial tissue and exploring the underlying mechanisms of disease pathogenesis. This study provides valuable insights into the landscape of DNA methylation research in RA and highlights the importance of epigenetics in autoimmune diseases.

## 1. Introduction

Rheumatoid Arthritis (RA) stands as an intricate autoimmune inflammatory ailment, inciting joint inflammation and emerging as the most prevalent variant of inflammatory arthritis, impacting approximately 0.5% to 1% of the global populace.^[1,2]^ Its occurrence registers 2 to 3 times higher among women in comparison to men.^[3]^ Familial factors contribute to the condition in 40% to 65% of seropositive patients and 20% of seronegative patients.^[4]^ The 2010 edition of RA diagnostic criteria comprises 4 facets: joint engagement, serological anomalies ascertained through RFs and ACPAs assessments, heightened indicators of acute inflammatory response, and arthritis duration.^[5]^ Notably, RF measurements usually yield negative results in early RA.^[6]^ Similarly, the ACPAs test exhibits low sensitivity in RA diagnosis.^[7]^ Consequently, the novel RA classification criteria exhibit their imperfections as well, underscoring the significance of identifying novel serological biomarkers with substantial clinical utility to enhance RA diagnosis. In terms of treatment, non-steroidal anti-inflammatory drugs, glucocorticoids, and disease-modifying anti-rheumatic drugs represent commonplace interventions for RA. However, they often accompany side effects and demonstrate limited efficacy.^[8,9]^ Although the precise etiology of RA remains elusive, it is generally attributed to a blend of epigenetic alterations, immune dysregulation, and environmental influences.^[10]^ DNA methylation emerges as the quintessential epigenetic modification in humans, intimately linked with immune dysregulation.^[11–13]^ Furthermore, RA-related DNA methylation patterns exhibit site-specific variances linked to immune pathways.^[14]^ Recognizing the potential reversibility of epigenetic alterations, probing epigenetic malfunctions in autoimmune disorders has the capacity to uncover novel biomarkers and innovative therapeutic targets. Bibliometric analysis, a systematic and quantitative technique for scrutinizing scientific literature, assumes a pivotal role in research endeavors. It expedites the comprehension of epigenetic trends within rheumatoid arthritis, facilitating the identification of eminent authors and prolific research institutions, while swiftly pinpointing high-confidence, compelling research areas.^[15]^ Herein, we undertake a visual analysis and synthesis of DNA methylation trends in rheumatoid arthritis, offering insights into the role of DNA methylation in the mechanisms, diagnostics, and treatments of this condition.

## 2. Materials and methods

### 2.1. Data source

**Figure 1.**
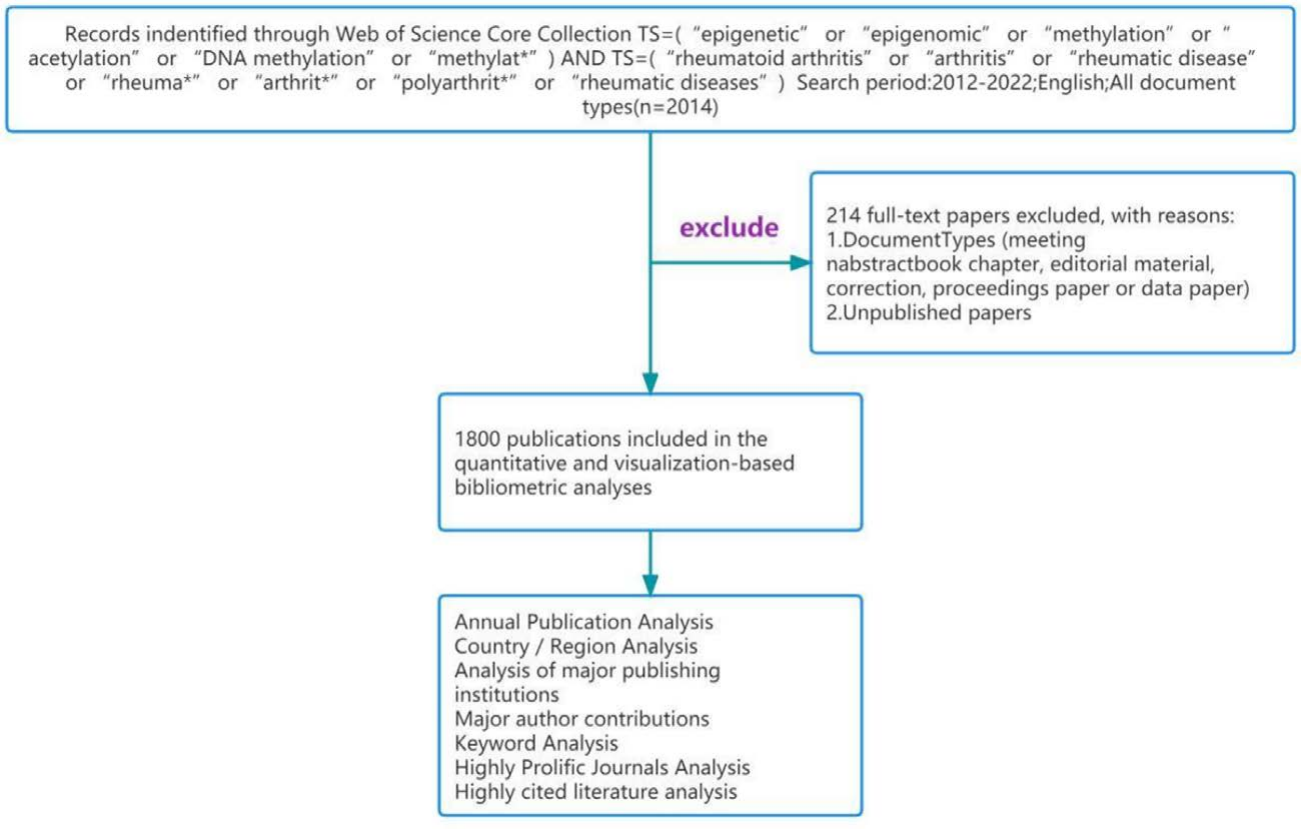
Flowchart for search process.

The source data for this investigation were obtained from the Web of Science core collection database, with publication dates restricted to the period from January 1, 2003, to December 31, 2022. We limited the article types to “Article” and “Review” exclusively. Two researchers independently conducted the retrieval process using the following search formula: TS=(“epigenetic” or “epigenomic” or “methylation” or “acetylation” or “DNA methylation” or “methylat *”) AND TS=(“rheumatoid arthritis” or “arthritis” or “rheumatic disease” or “rheuma * “Or” arthrit * “or” polyarthrit * “or” rheumatic diseases “) as of April 20, 2023. We restricted the language to English. A total of 2014 articles were retrieved, and 214 articles that did not meet the criteria were excluded. Finally, 1800 valid articles were downloaded in plain text format and saved as “download *,” as depicted in Figure 1.

For visual analysis, we employed CiteSpace (version 6.1.6R),^[16,17]^ VOSviewer (version 1.6.18), and COOC (version 13.7) to facilitate data visualization.^[18,25]^ Bibliometric analysis, encompassing clustering, co-occurrence, co-citation, and coauthor analyses,^[19]^ was employed as the principal analytical approach. The most frequently employed metric in this study was co-citation, which highlights the influence of cited articles.^[20–22]^ We collected fundamental data including authorship, institutional affiliations, geographical locations, journals, keywords, and references. CiteSpace served as our tool for conducting author, reference, and journal analyses. Additionally, we explored citing bursts in literature and the recurrence of new references and keywords.^[23]^ The interplay between citing and cited journals was depicted using a dual-map overlay of journals. Parameters were set as follows: the timeframe was selected from 2003 to 2022 with a yearly interval of 1, and all options in the term source were chosen sequentially, followed by node type selection and criteria (k = 25). In the graph, each node represents an observation, with node size denoting frequency—larger nodes indicating higher frequencies. Connections between nodes signify collaboration, co-occurrence, or co-reference relationships. Node colors correspond to different years; colors from inner to outer circles represent the years from 2003 to 2022. VOSviewer, another bibliometric software, was employed for creating and visualizing bibliometric maps. This software provides a summary table interface and conducts a comprehensive, in-depth bibliometric analysis. It differs from conventional bibliometric procedures^[24]^ in its emphasis on graphical representations of bibliometric data. Each node on the VOSviewer map corresponds to a specific parameter, such as country/region, journal, or keyword, and node sizes are determined by properties such as the number of publications, frequency of appearance, or citation counts. Nodes and lines are categorized into clusters based on the clusters they belong to, determining their colors. Connections are depicted as lines connecting nodes. COOC, an emerging bibliometric software, complements the analyses conducted using CiteSpace and VOSviewer by providing additional measurement results.^[25]^

## 3. Results

### 3.1. Analysis of annual publications

**Figure 2.**
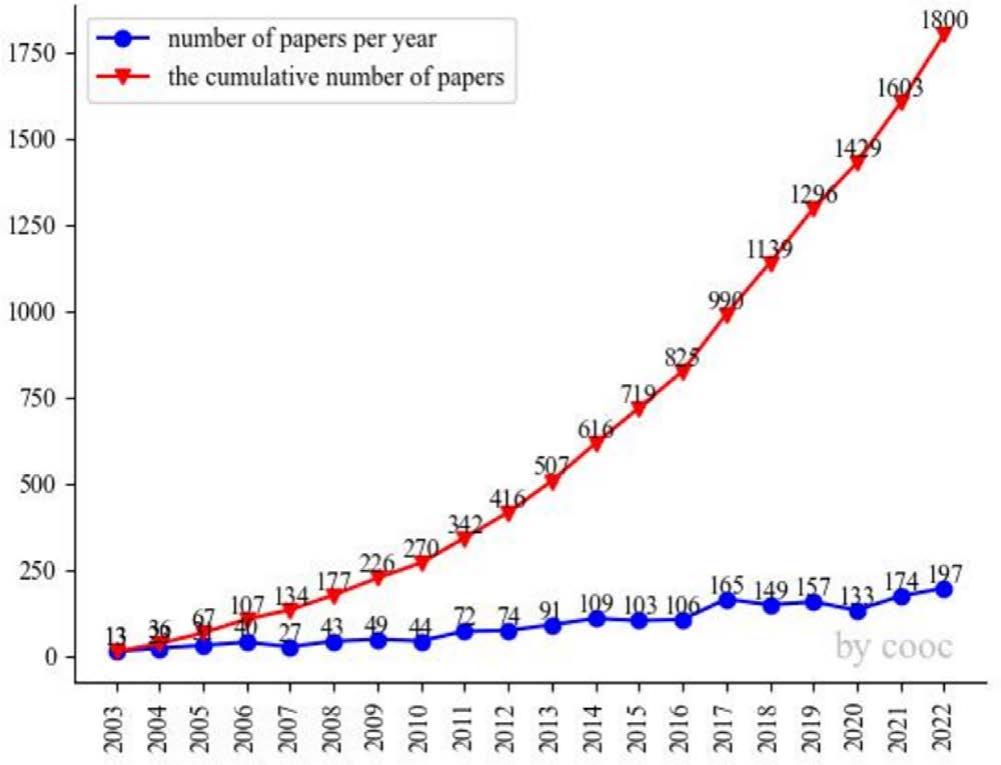
Annual publications of DNA methylation in RA from 2003 to 2022.

In this study, we analyzed a total of 1800 articles related to DNA methylation in the context of rheumatoid arthritis. We conducted a visual examination of publication trends using Co-Occurrence 12.9,^[25]^ as shown in Figure 2. The analysis of DNA methylation in rheumatoid arthritis revealed a consistent upward trajectory, reaching its peak of 197 articles in 2022. This trend indicates sustained and growing interest in this research area.

### 3.2. National/regional analysis

Our statistical analysis identified 70 countries actively participating in research on rheumatoid arthritis and DNA methylation from 2003 to 2022. Table 1 presents the top 10 countries with the highest number of articles, with the United States leading with over 500 articles. China closely follows with a substantial number of articles, and other countries in the range of 100 to 300 articles include the United Kingdom, Italy, Germany, the Netherlands, and Japan. This demonstrates the significant contribution of the United States to the field, highlighting the importance of DNA methylation in the diagnosis and treatment of rheumatoid arthritis.

**Table 1:** Top 10 productive countriesregions related to DNA methylation in RA.

Rank	Country	Count	Centrality	TLS
1.	USA	511	0.33	396
2.	CHINA	377	0.04	122
3.	ENGLAND	190	0.18	228
4.	ITALY	124	0.22	117
5.	GERMANY	119	0.2	167
6.	NETHERLANDS	102	0.1	144
7.	JAPAN	100	0.09	68
8.	SWITZERLAND	78	0.09	107
9.	FRANCE	76	0.22	83
10.	SPAIN	74	0.05	66

**Figure 3.**
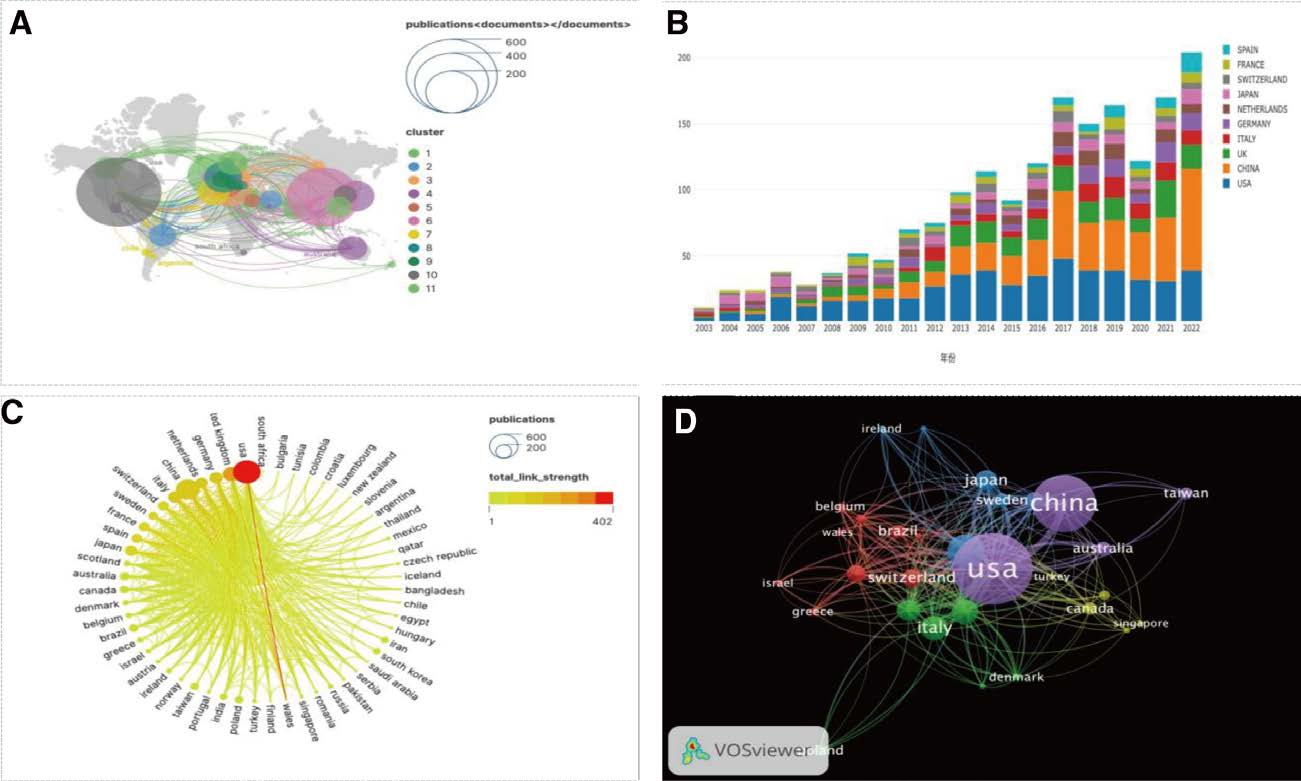
(A) Geographic distribution map based on the total number of publications in different country regions (B) Trends in annual publications in the top ten countries or regions from 2003 to 2022 (C) A map of international collaborations visualization by country or region (D) Citation network visualization maps for countries and regions generated using the VOS viewer. The thickness of the lines reflects the strength of the citations.

Figure 3A, a global publication map, reveals that research on DNA methylation primarily originates from Asia, North America, and various European countries. Figure 3B depicts the annual publication counts in these countries, with China showing the highest annual growth rate, closely followed by Germany.

Figure 3C illustrates international collaboration, with the United States extensively cooperating with numerous countries. The United Kingdom and the United States emerge as prominent collaborative partners, while other countries exhibit lower levels of cooperation. Figure 3D shows the total link strength (TLS) is indicated by the thickness of connecting lines between nodes, with the United States having the highest TLS value (TLS = 396), followed by the United Kingdom, Germany, Northern Ireland, and China.

### 3.3. Analysis of the main publishing institutions

**Figure 4.**
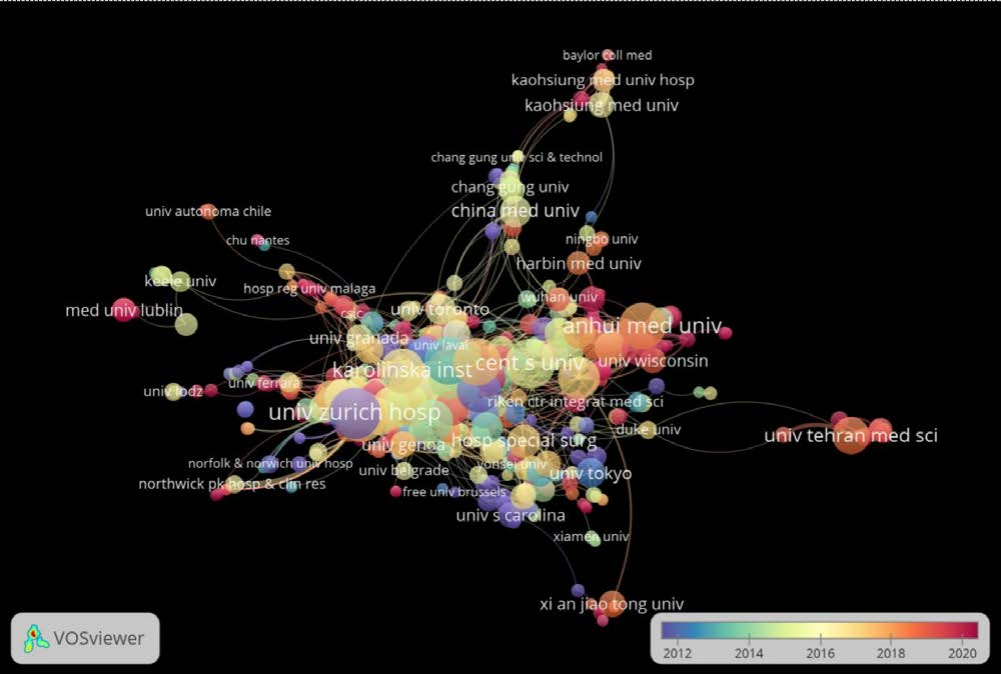
Visualization map of institutional cooperation networks generated by VOSviewer software.

A total of 2139 organizations participated in research related to rheumatoid arthritis and DNA methylation from 2003 to 2022. Table 2 presents the top ten prolific institutions, with the United States, the United Kingdom, and China having multiple research-engaged institutions. University Hospital Zurich, University College Oxford, Central South University, University of California San Diego, and University of Michigan were the top 5 institutions contributing the most research articles. These institutions collectively accounted for 17.8% of the total publications. University Hospital Zurich had the highest number of citations, while the University of California San Diego was the most collaborative institution. Figure 4, visualized using VOSviewer, illustrates collaborative relationships among institutions. University Hospital Zurich, University College Oxford, and Karolinska Institutet had the highest TLS values, indicating extensive collaboration within organizations.

**Table 2:** The top 10 productive institutions ranked by the numbers of publications.

Rank	Institutions	Count	CEntrality	TLS	Citations
1.	Univ Zurich Hosp	39	0.03	80	2983
2.	Univ Oxford	37	0.05	73	2068
3.	Cent s Univ	36	0.03	39	2175
4.	Univ Calif San Diego	33	0.08	61	2066
5.	Univ Michigan	33	0.02	53	2256
6.	Anhui Med Univ	32	0.03	38	782
7.	Karolinska Inst	30	0.06	69	1577
8.	Harvard Univ	28	0.07	67	2470
9.	Univ Sao Paulo	27	0.01	29	1018
10.	Fudan Univ	26	0.05	43	819

### 3.4. Author analysis with co-cited authors

**Figure 5.**
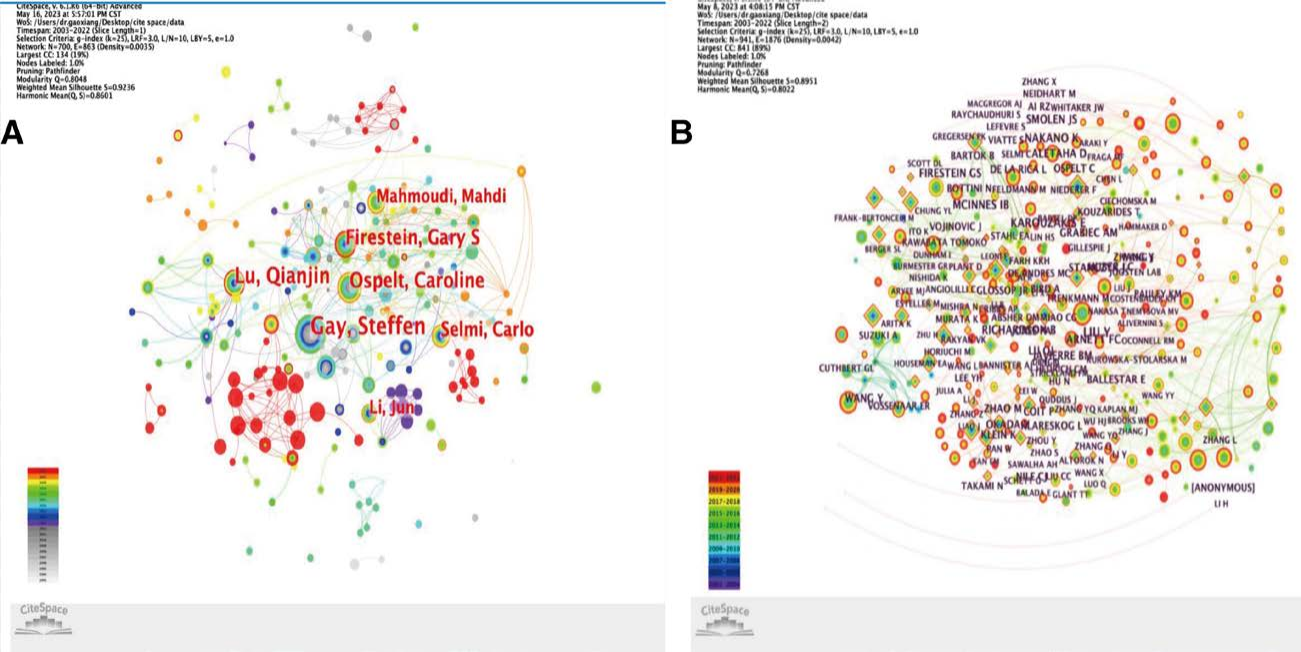
(A) Visualization of the author partnership network generated by Citespace software (B) Co-cited author network visualization graph generated by Citespace software.

The study covered 23,365 authors and 58,636 co-cited authors from 2011 to 2021. Figure 5 shows the visualization of the author network. The clustering performance of the network increases as the value grows. The homogeneity of the network is measured by another indicator, silhouette value. The centrality of a node can be determined by its betweenness centrality (BC), which shows the importance of a node in the network. Table 3 lists the 20 most prolific authors, with Gay Steffen, Lu QJ, and Ospelt C being the top 3. The authors exhibited relatively low centrality, with Gay Steffen having the highest betweenness centrality value of 0.04. The modular Q value reached 0.8048, indicating substantial network homogeneity and clustering effects. Table 4 presents the top 20 co-cited authors, with Karouzakis, E, Liu, Y, and Nakano, K being the most frequently cited authors, each with more than 100 citations.

**Table 3:** The top 20 most productive authors related to DNA methylation in RA.

Rank	Author	Publications	Centrality	Rank	Author	Publications	Centrality
1	Gay, Steffen	37	0.04	11	Sawalha, Amr H	11	0.01
2	Lu, Qianjin	27	0.01	12	Karouzakis, Emmanuel	11	0
3	Ospelt, Caroline	22	0.02	13	Zhao, Ming	11	0
4	Firestein, Gary S	21	0.02	14	Guo, Shicheng	11	0
5	Selmi, Carlo	17	0	15	Klein, Kerstin	10	0.01
6	Li, Jun	14	0	16	Aslani, Saeed	10	0
7	Mahmoudi, Mahdi	14	0	17	Thompson, Paul R	10	0
8	Wang, Wei	13	0	18	Wu, Haijing	9	0.01
9	Gay, Renate E	13	0	19	He, Dongyi	9	0
10	Ballestar, Esteban	12	0.03	20	Alarcon-riquelme, Marta E	9	0.02

**Table 4:** The top 20 most productive co-cited authors related to DNA methylation in RA.

Co-ciation	TLS	Rank	Co-cited author	Co-ciation	TLS
306	1928	11	Stanczyk, J	161	1028
243	1064	12	Okada, Y	154	544
213	1362	13	Coit, P	151	896
212	1193	14	Huber, LC	142	723
202	558	15	Selmi, C	138	701
196	1590	16	Klein, K	127	674
184	516	17	Javierre, BM	117	726
178	984	18	Klareskog, L	117	490
164	1038	19	Glossop, JR	116	680
161	567	20	Hedrich, CM	110	718

### 3.5. Keyword analysis

**Figure 6.**
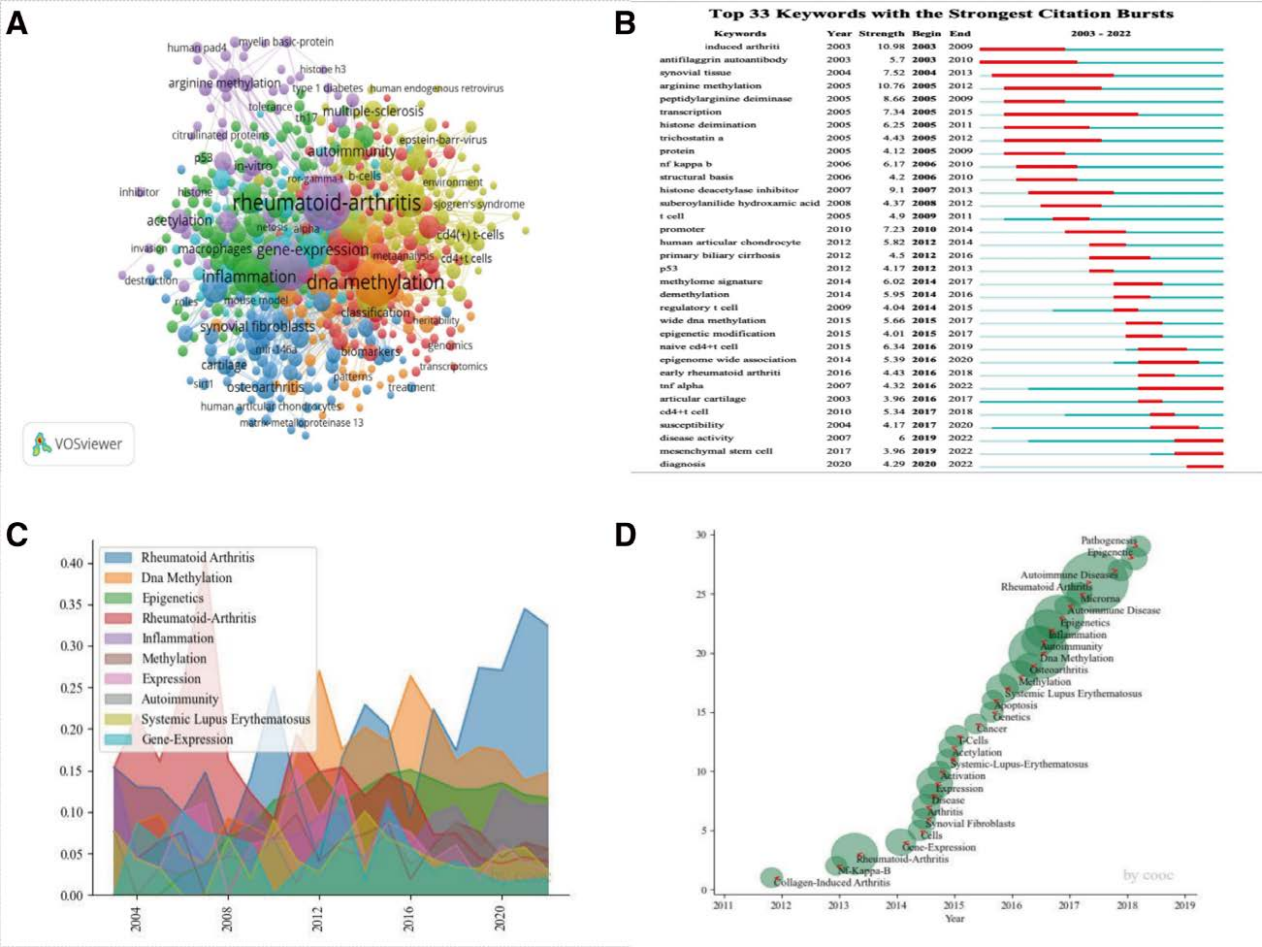
(A) Keyword network visualization graph generated by VOSviewer software (B) Keyword citation bursts generated by Citespace software (C) Year-to-year attention change graph generated by COOC software (D)Weighted topic evolution path graph generated by COOC software.

Keyword analysis is crucial for understanding research themes and directions.Out of 7464 extracted keywords, 724 met the threshold for inclusion. Figure 6A illustrates keywords grouped into 6 clusters, with “rheumatoid arthritis,” “DNA methylation,” and “expression” being frequently encountered terms. Table 5 lists the top 20 keywords with the highest frequency related to DNA methylation in RA. Figure 6B shows the keyword outbreaks, with “epigenome-wide association” being the most cited keyword in 2020. Terms like “tumor necrosis factor,” “disease activity,” “mesenchymal stem cell,” and “diagnosis” have consistently gained attention. COOC analysis traced the trajectory of annual attention and research hotspots. “Rheumatoid arthritis” gained increasing attention in recent years, while “DNA methylation” maintained consistently high attention, as shown in Figure 6C. “Epigenetic,” “pathogenesis,” “autoimmune disease,” and “rheumatoid arthritis” are current research hotspots, as shown in Figure 6D,

**Table 5:** The top 20 keywords with the highest frequency related to DNA methylation in RA.

Rank	Keywords	Counts	Central	Rank	Keywords	Counts	Central
1.	Rheumatoid arthriti	980	0.06	11.	Association	126	0.02
2.	DNA methylation	469	0.04	12.	NF kappa b	120	0.04
3.	Expression	350	0.01	13.	Methylation	116	0.05
4.	Systemic lupus erythematosus	275	0.02	14.	Inflammation	109	0.04
5.	Gene expression	222	0.06	15.	Synovial fibroblast	101	0.04
6.	T cell	196	0.05	16.	Collagen induced arthriti	99	0.08
7.	Disease	144	0.05	17.	Arthriti	89	0.03
8.	Cell	140	0.03	18.	Risk	87	0.03
9.	Autoimmune disease	131	0.05	19.	Gene	84	0.01
10.	Activation	126	0.05	20.	Histone deacetylase inhibitor	84	0.04

### 3.6. Distribution of source journals and top 10 highly cited articles

**Figure 7.**
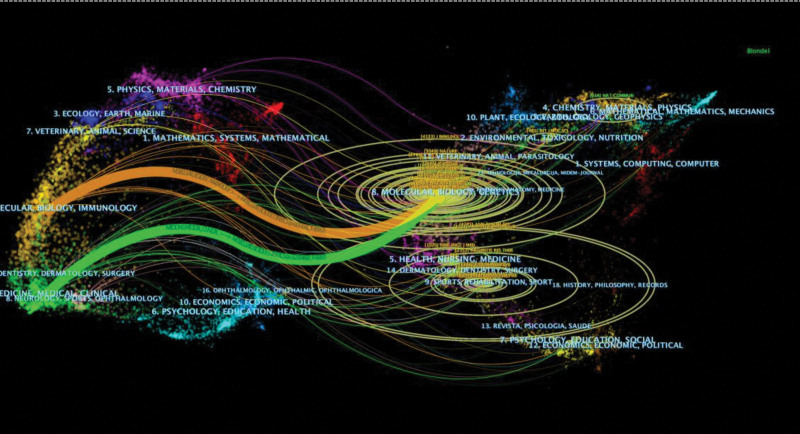
Dual-map overlay of journals related to DNA methylation in RA.

Out of 1800 articles, 615 academic journals published the research. Table 6 lists the top 10 journals with the highest number of articles, with ADV EXP MED BIOL and AM J HUM GENET exceeding 100 articles. These journals, along with AM J PATHOL, were the most prolific in epigenetics within rheumatoid arthritis research. Notably, 80% of the top 10 journals are based in the United States. Table 7 presents the most frequently cited journals among the top 10 co-cited journals, with ARTHRITIS RHEUM being the highest cited. Table 8 showcases the top 10 research studies focusing on DNA methylation and rheumatoid arthritis, with the Embo Journal study from 2003 having the most citations. The study by Kalliolias, Gd, et al, published in Nature Reviews Rheumatology in 2016, received significant attention. Figure 7, the Journal Stack Diagram, illustrates how research topics are distributed among journals. Different colored lines represent reference connections between journals.

**Table 6:** Top10 journals related to the research of DNA methylation in RA.

Rank	Journal	Count	Centality	IF	SCI	Country
1.	ADV EXP MED BIOL	115	0.3	3.65	Q2	USA
2.	AM J HUM GENET	91	0.13	11.04	Q1	USA
3.	AM J PATHOL	87	0.14	5.77	Q1	USA
4.	ARTHRITIS RHEUM	87	0.14	15.438	Q1	USA
5	ANN RHEUM DIS	72	0 05	27 97	Q1	UK
6.	ACTA PHARMACOL SIN	66	0.11	7.17	Q1	China
7.	ACS CHEM BIOL	56	0.08	4.63	Q2	USA
8.	ADV IMMUNOL	53	0.06	5.32	Q2	USA
9.	AM J MED	31	0.06	5.93	Q1	USA
10.	AM J RESP CRIT CARE	30	0.03	30.53	Q1	USA

**Table 7:** Top ten co-cited journals related to the research of DNA methylation in RA.

Rank	Co-cited Journal	Citations	IF	SCI	Country
1.	ARTHRITIS RHEUM	6591	15.438	Q1	USA
2.	ANN RHEUM DIS	4289	27.97	Q1	UK
3.	J IMMUNOL	4129	5.43	Q2	USA
4.	NATURE	3054	69.50	Q1	UK
5.	P NATL ACAD SCI USA	2786	18.10	Q1	USA
6.	ARTHRITIS RES THER	2362	5.61	Q1	UK
7	J BIOL CHEM	2543	5 49	Q2	USA
8.	J AUTOIMMUN	1974	19.90	Q1	Netherlands
9.	CELL	2052	66.85	Q1	USA
10.	PLOS ONE	2098	3.75	Q2	USA

**Table 8:** The top ten DNA methylation in RA-related original articles with the most citations.

Title	Year	Journal	Institutions	citation	Author	Main conclusion
Monocytic Cells Hyperacetylate Chromatin Protein Hmgb1 to Redirect it Towards Secretion	2003	Embo Journal	San Raffaele Sci Inst	1041	Bonal di, T	Monocytes and macrophages acetylate hmgb1 extensively upon activation with lipopolysaccharide; moreover, forced hyperacetylation of hmgb1 in resting macrophages causes its relocalization to the cytosol. cytosolic hmgb1 is then concentrated by default into secretory lysosomes, and secreted when monocytic cells receive an appropriate second signal.
Inflammation in Osteoarthritis	2011	Current Opinion In Rheumatol ogy	Hosp Special Surg	943	Goldri ng, Mb	novel stress-induced and proinflammatory mechanisms underlying the pathogenesis of osteoarthritis, with particular attention to the role of synovitis and the contributions of other joint tissues to cellular events that lead to the onset and progression of the disease and irreversible cartilage damage
Tnf Biology, Pathogenic Mechanisms and Emerging Therapeutic Strategies	2016	Nature Reviews Rheumatol ogy	Hosp Special Surg	716	Kallioli as, Gd	we present molecular mechanisms underlying the roles of tnf in homeostasis and inflammatory disease pathogenesis, and discuss novel strategies to advance therapeutic paradigms for the treatment of tnf-mediated diseases.
Pesticides and Human Chronic Diseases	2013	Toxicology And Applied	Univ Tehran Med Sci	703	Mostaf alou, S	we present the highlighted evidence on the association of pesticide exposure with the incidence of chronic diseases and introducemost citations.
Evidences, Mechanisms, and Perspectives		Pharmacol ogy				genetic damages, epigenetic modifications, endocrine disruption, mitochondrial dysfunction, oxidative stress, endoplasmic reticulum stress and unfolded protein response, impairment of ubiquitin proteasome system, and defective autophagy as the effective mechanisms of action.
Epigenome-Wid e Association Data Implicate DNA Methylation as an Intermediary of Genetic Risk In Rheumatoid Arthritis	2013	Nature Biotechnol ogy	Karolinska Inst	674	Liu, Y	DNAmethylation is a potential mediator of genetic risk in rheumatoid arthritis
Duality of Fibroblast-Like Synoviocytes In Ra: Passive Responders and Imprinted Aggressors	2013	Nature Reviews Rheumatol ogy	Ucsd Sch Med	649	Bottini , N	the molecular bases of this “imprinted aggressor” phenotype are being clarified through genetic and epigenetic studies. the dual behavior of fls in ra suggests that fls-directed therapies could become a complementary approach to immune-directed therapies in this disease.
Physiology and Pathophysiolog y of Matrix Metalloprotease s	2011	Amino Acids	Univ Groningen	562	Klein, T	we will give an overview of 23 members of the human mmp family and describe functions, linkages to disease and structural and mechanistic features. mmps can be grouped into soluble (including matrilysins) and membrane-anchored species. we adhere to the “mmp nomenclature” and provide the reader with reference to the many, often diverse, names for this enzyme family in the introduction.
The Nest Long Ncrna Controls Microbial Susceptibility and Epigenetic Activation of the Interferon-Gam ma Locus	2013	Cell	Stanford Univ	525	Gome z, Ja	Thus, this incrna regulates epigenetic marking of ifn-gamma-encoding chromatin, expression of ifn-gamma, and susceptibility to a viral and a bacterial pathogen.

### 3.7. Analysis of the reference

**Figure 8.**
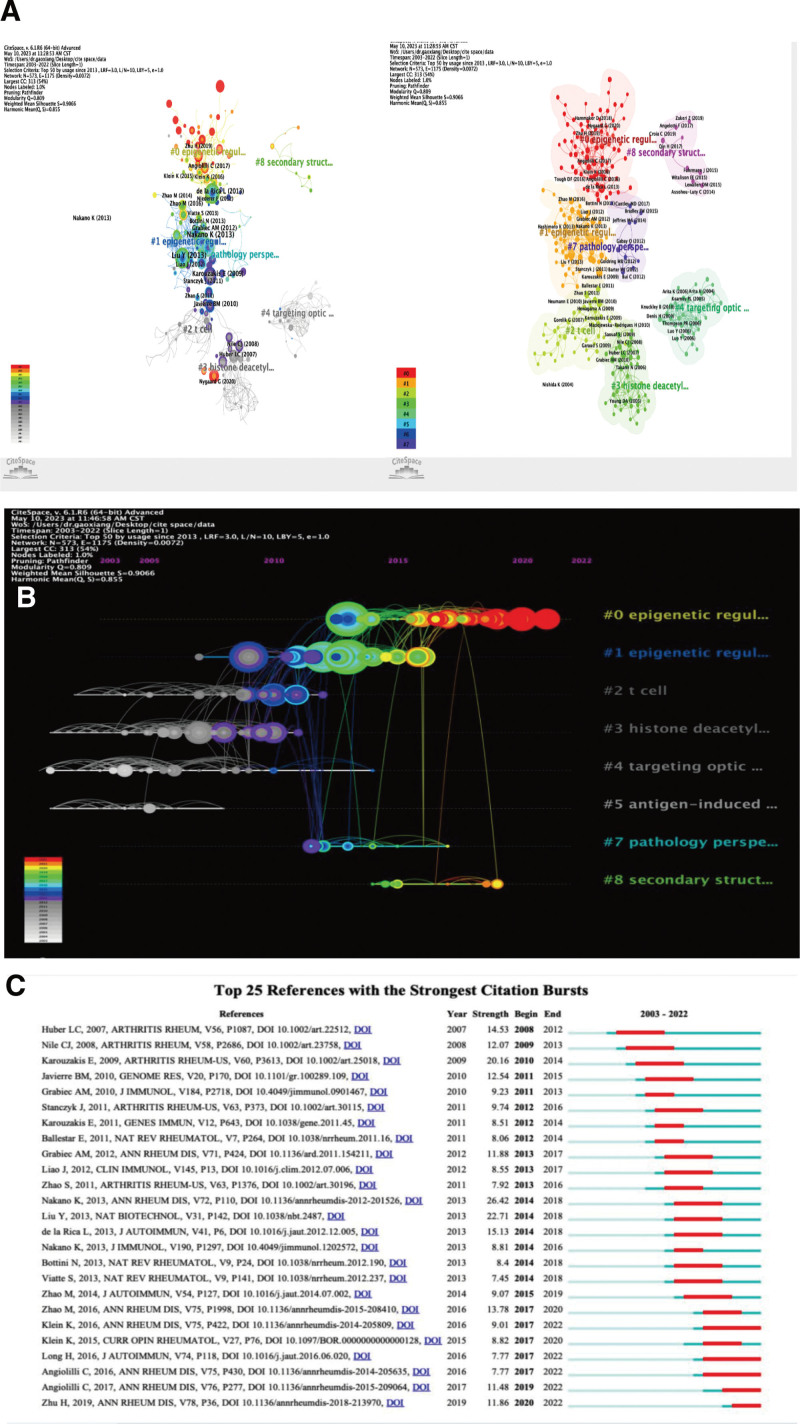
(A) Reference sharing and clustering map generated by Citespace software (B) Reference citation time zone map generated by Citespace software (C).Reference citation explosion graph generated by Citespace software.

A total of 88,125 references were cited. Table 9 presents the top ten references for studies related to DNA methylation in rheumatoid arthritis. The top 10 journals, based on impact factors, included Ann Rheum Dis, Nat Biotechnol, and Genome Res. Figure 8A displays a mixed network graph of co-occurring reference clusters, with strong clustering effects. Figure 8B offers a chronological view of co-cited references, indicating evolving research hotspots. Figure 8C highlights the top 25 references with prominent citation bursts, emphasizing the enduring significance of DNA methylation in rheumatoid arthritis research.

**Table 9:** Top ten cited references concerning the research of DNA methylation in RA.

Rank	Journal	Title	Citation	IF (2020)	Year	Author
1	Ann Rheum Dis	DNA methylome signature in rheumatoid arthritis	148	16.102	2013	Kazuhisa Nakano
2	Nat Biotechnol	Epigenome-wide association data implicate DNA methylation as an intermediary of genetic risk in rheumatoid arthritis	133	36.702	2013	Yun Liu
3	ARTHRITIS RHEUM	DNA hypomethylation in rheumatoid arthritis synovial fibroblasts	125	9.59	2009	Emmanuel Karouzakis
4	J Autoimmun	Identification of novel markers in rheumatoid arthritis through integrated analysis of DNA methylation and microRNA expression	124	7.139	2013	Lorenzo de la Rica

## 4. Discussion

### 4.1. Basic profile of DNA methylation in rheumatoid arthritis

A total of 1800 pertinent articles were retrieved in this study. In recent years, there has been a growing interest among researchers in unraveling the molecular mechanisms underlying rheumatoid arthritis, leading to the emergence of a prolific group of authors, including prominent names such as Gay Steffen, Lu Qianjin, and Ospelt Caroline. In terms of national and regional contributions, the United States takes the lead in this field, with University Hospital Zurich being a prolific publisher of related studies. The most frequently cited authors and references often center on epidemiological aspects and the characteristics of rheumatoid arthritis. The top 4 keywords that are both highly frequent and prominent include “rheumatoid arthritis,” “DNA methylation,” “expression,” and “gene expression.” Emerging research hotspots are indicated by keywords such as “diagnosis,” “pathogenesis,” “epigenetic,” “DNA methylation,” “rheumatoid arthritis,” and “autoimmune disease.” These keywords shed light on the evolving trends and emerging areas of interest in the study of rheumatoid arthritis and DNA methylation.

### 4.2. Hotspot analysis of DNA methylation in rheumatoid arthritis

This study highlights the current research hotspots in the field of rheumatoid arthritis, which include “epigenetic regulation,” “pathogenesis,” and “epigenome-wide association.” Among various epigenetic modifications, DNA methylation stands out as the most extensively studied due to its numerous advantages over other epigenetic modifications. Notably, DNA methylation can be stably inherited through multiple cell divisions.^[26]^ This process is mediated by a family of enzymes known as DNA methyltransferases (DNMT).^[27]^ In humans, DNA methylation primarily occurs within CpG islands, which are regions rich in cytosine-phosphate-guanine dinucleotide (CpG) sites. Approximately 60% to 70% of annotated gene promoters are associated with CpG islands, making the methylation of these islands pivotal in regulating gene expression.^[15]^Numerous studies have explored DNA methylation-targeting drugs, such as 2-deoxy-5-azacitidine, decitabine, and zebularine,^[28–30]^ in the context of treating various types of cancer and rheumatic diseases. Some research suggests that 5’-AZA-mediated demethylation of the interleukin-10 (IL-10) gene may inhibit the development of RA by enhancing the production of the immunosuppressive IL-10.^[31]^ Additionally, a novel DNMT inhibitor called SGI-1027, a lipophilic, quinoline-based compound, has been identified. It causes the degradation of DNMT1 and demethylation of genes such as cyclin-dependent kinase inhibitor 2A, MutL homolog 1 (MLH1), and tissue inhibitor matrix metalloproteinase 3.^[32]^ On another note, the DNA methyltransferase activator budesonide has shown promise in increasing DNA methylation levels and improving tender and swollen joint counts in RA patients.^[33]^Turning to the aspect of pathogenesis, recent research has uncovered several potential triggers for rheumatoid arthritis. Substantial evidence points to smoking as a causative factor for seropositivity in RA and RA-related interstitial lung disease.^[34,35]^Furthermore, not only inhalation of cigarette smoke but also exposure of the respiratory tract to various factors, including asthma,^[36]^ COVID-19,^[37]^ as well as environmental factors such as coal dust, silica, industrial particles,^[38]^ and air pollutants from traffic,^[39]^ can contribute to the development of RA. Diet also emerges as a significant factor, with studies indicating that the gut epithelium and the gut microbiota play critical roles in maintaining barrier function and inflammation in the gut and colon.^[40–42]^Angelica polysaccharide has been found to modulate the intestinal microbiota and alter the expression of genes such as Cldn5, Slit3, and Rgs18, ultimately improving RA.^[43]^

Regarding epigenome-wide association studies (EWAS), RA is recognized as a complex autoimmune disease.^[44]^ These studies have underscored the influence of cellular phenotypes on the etiology and outcomes of complex diseases, with genomic DNA modification through epigenetic mechanisms playing a pivotal role in these effects.^[45,46]^ EWAS can mitigate the risk of false positives often encountered in studies and reveal significant regulatory relationships between the epigenome and RA through the analysis of large-scale methylation quantitative trait loci.^[47]^ Furthermore, EWAS can identify common apparent mutations in disease by detecting the distribution of methyl groups. For instance, changes in the pathogenic activity of B cells attributed to DNA methylation are strongly associated with RA.^[48]^

### 4.3. Hotspots and emerging frontiers of DNA methylation in RA

Analyzing citation trends, we have identified 2 keywords, “diagnosis” and “mesenchymal stem cells,” that experienced a surge in citations from 2020 to 2022.Diagnosis: DNA methylation has gained prominence as a potential indicator for diagnosing RA. Timely diagnosis and treatment are critical for improving the long-term clinical outcomes of RA patients. A study by RazaK et al demonstrated that early treatment within 12 weeks of disease onset significantly improved outcomes compared to delayed treatment, resulting in a 30% reduction in the latter group.^[49]^ LairdP. W et al observed DNA methylation changes occurring in the early stages of the disease, suggesting its utility as an early warning indicator.^[50]^

Targeted methylation sequencing technology has been employed to assess the methylation status of specific CpG sites, including those in the differentially methylated region of HIPK3 in 235 recruited RA patients.^[51]^ The results indicate that HIPK3 can serve as a new diagnostic marker for RA. Combining HIPK3 with anti-citrullinated protein antibody and rheumatoid factor (RF+) in a prediction model achieved a higher AUC of 0.864.^[52]^ JiananZhao et al identified significant hypermethylation of the HTR2A promoter region in peripheral blood samples from RA patients, suggesting a potential diagnostic role in rheumatoid arthritis.^[52]^ GangFang et al identified APOL6 and CCDC88C as potential novel biomarkers of RA, as they are associated with differential methylation sites and play crucial roles in the disease progression.^[53]^ Additionally, serum circulating miRNA-5196 has shown promise as a biomarker for predicting positive treatment outcomes of TNF-α treatment in RA and ankylosing spondylitis patients.^[54,55]^ StanczykJ et al discovered enhanced expression of miRNA-203 in RAFLS, which interacts with hypomethylation of the MMP1 and IL-6 gene promoters, suggesting a potential link between miRNA and DNA methylation in RA pathogenesis.^[56]^

Mesenchymal stem cells (MSCs): Research involving MSCs and their relationship with RA has yielded significant results since 2018. MSCs exhibit multiple functions, including promoting tissue regeneration and immune regulation through their extracellular vesicles.^[57]^ They can modulate T cell proliferation and differentiation and polarize toward an anti-inflammatory phenotype, reducing the production of proinflammatory factors.^[58,59]^ The use of MSCs in treating RA has shown considerable promise, with studies confirming both safety and significant efficacy.^[60,61]^

However, it worth noting that some studies have raised concerns about the potential immunosuppressive effects of MSCs, which may increase the risk of tumor development in patients.^[62,63]^ This aspect is still under investigation, and further research is needed to fully understand the implications of MSC-based therapies in RA.

## 5. Conclusion

The study on the association between DNA methylation and rheumatoid arthritis, conducted through bibliometric analysis and the exploration of international collaborations, authors, publications, keywords, and research hotspots, offers valuable insights to the medical community. It illuminates the evolving landscape of DNA methylation research in the context of rheumatoid arthritis and underscores the significance of epigenetics in the study of autoimmune diseases.

As genetic technology continues to advance, research aimed at diagnosing and treating autoimmune diseases is expected to become more sophisticated. However, it essential to acknowledge that investigating the relationship between DNA methylation and rheumatoid arthritis faces various challenges. The clinical application of epigenetic drugs designed to regulate abnormal DNA methylation patterns in RA is still in its infancy. While the FDA has approved blood tests based on DNA methylation biomarker technology for colorectal cancer screening,^[64]^ similar breakthroughs are eagerly anticipated in rheumatoid arthritis research.

In the near future, the hope is to discover noninvasive DNA methylation biomarkers that can be readily employed in daily medical practice for the early detection of RA, potentially before irreversible joint damage occurs. This not only promises a deeper understanding of how the epigenetic landscape contributes to the pathogenesis of RA but also opens doors to leveraging DNA methylation mechanisms for the diagnosis and treatment of the disease. Such advancements could not only slow or mitigate disease progression but also lead to overall reductions in healthcare costs and a decrease in the societal and patient burden associated with rheumatoid arthritis.

### 5.1. Strengths and limitations

This study presents the first-ever bibliometric analysis of rheumatoid arthritis and DNA methylation. By employing systematic search and quantitative statistical analysis, our research provides a comprehensive and visually informative overview of the literature in this field, going beyond the confines of a traditional literature review. However, our study is not without its limitations.

While the Web of Science core collection database is extensive and encompasses a significant portion of scholarly articles, it may not include every pertinent publication on this topic. There could be research articles, reviews, or other forms of literature in alternative databases or sources that our analysis did not incorporate. Consequently, there is a chance that our study may have missed valuable contributions to this field.

Future research in this area should prioritize expanding the scope of analysis, possibly by integrating additional databases or sources. This approach would ensure a more comprehensive overview of the subject matter, thereby augmenting the accuracy and completeness of bibliometric analyses in this field.

## Acknowledgments

We thank the web of science Core Collection for providing the raw data for this study.

## Author contributions

**Conceptualization:** Changhua Liu.

**Software:** Longxiang Huang.

**Visualization:** Longxiang Huang.

**Writing – original draft:** Xin Huang.

**Writing – review & editing:** Xiang Gao.
